# Menopause age, reproductive span and hormone therapy duration predict the volume of medial temporal lobe brain structures in postmenopausal women

**DOI:** 10.1016/j.psyneuen.2023.106393

**Published:** 2023-12

**Authors:** Jessica J. Steventon, Thomas M. Lancaster, Emily Simmonds Baker, Matthew Bracher-Smith, Valentina Escott-Price, Katherine S. Ruth, William Davies, Xavier Caseras, Kevin Murphy

**Affiliations:** a Neuroscience and Mental Health Research Institute, Cardiff University, UK; b Department of Psychology, University of Bath, UK; c MRC Centre for Neuropsychiatric Genetics and Genomics, Division of Psychological Medicine and Clinical Neurosciences, Cardiff University, UK; d University of Exeter Medical School, RILD Level 3 Royal Devon & Exeter Hospital, Barrack Road, Exeter EX2 5DW, UK; e Cardiff University Brain Research Imaging Centre (CUBRIC), Cardiff University, UK; f School of Physics and Astronomy, Cardiff University, UK

**Keywords:** Menopause, Oestrogen, Hormone replacement therapy, Imaging, Alzheimer, Dementia

## Abstract

Medial temporal lobe (MTL) atrophy is correlated with risk and severity of Alzheimer disease (AD) pathology and cognitive decline. Increasing evidence suggest that oestrogens affect the aging of MTL structures. Here we investigate the relationship between reproductive hormone exposure, polygenic scores for AD risk and oestradiol concentration, MTL anatomy and cognitive performance in postmenopausal women. To this end, we used data from 10,924 female participants in the UK Biobank from whom brain MRI and genetic data were available. We fitted linear regression models to test whether the volume of structures comprising the MTL were predicted by a) timing related to menopause, b) the use and timing of hormone replacement therapy (HRT) and c) polygenic scores for AD risk and oestradiol concentration. Results showed that longer use of HRT was associated with larger parahippocampal volumes (2.53 mm^3^/year, p = 0.042). A later age of natural menopause, and a longer reproductive span, was associated with larger hippocampal (6.08 and 5.72 mm^3^/year, p = 0.0006 and 0.0005), parahippocampal (4.17 mm^3^ and 4.19 mm^3^/year, p = 0.00006 and 0.00001), amygdala (2.10 and 2.22 mm^3^/year, p = 0.028 and 0.01) and perirhinal cortical (2.56 and 2.95 mm^3^/year, p = 0.028 and 0.008) volumes. Superior prospective memory performance was associated with later age at natural menopause, and a longer reproductive span (ß = 0.05 and 0.05 respectively, p = 0.019 and 0.019). Polygenic scores for AD risk and for oestradiol concentration were not associated with MTL volume and did not interact with menopause-related factors to affect MTL structure. Our results suggest that HRT use did not have any detrimental effects on cognition or brain structure, whilst greater exposure to reproductive hormones across time is associated both with slightly larger volumes of specific MTL structures and marginally superior memory performance, independent of genetic risk for AD and genetic predisposition for higher oestradiol levels. However, the clinical utility of maintenance of oestrogens post-menopause for brain health and protection against cognitive decline is curtailed by the small effect sizes observed.

## Highlights

• Later menopause age was associated with larger volume in temporal brain structures.
• Longer reproductive span was associated with larger brain temporal volumes.
• Later menopause age was associated with better prospective memory.
• Longer reproductive span was associated with better prospective memory.
• Hormone Replacement therapy was weakly associated with parahippocampal volume.

## 1 Introduction

Longevity is increasing globally, and thus postmenopausal women are exposed to longer periods of depleted oestrogens. Evidence exists that levels of oestrogens are implicated in brain’s neural function and cytoarchitecture, and in vulnerability for neuropsychiatric conditions (Cutter et al., 2003). However, the effect of loss of oestrogens during menopause, or its maintenance through hormone-replacement therapy, on the brain and the health of aging women is still poorly understood.

Research has shown that events affecting lifetime exposure to oestrogens affect cognition and increase the risk for dementia in women. For example, bilateral oophorectomy in humans before menopause – that causes an abrupt decline of oestrogens – is associated with increased risk for cognitive decline and dementia (Rocca et al., 2007, Rocca et al., 2014). Further, the risk for cognitive decline increased with younger age of oophorectomy (Rocca et al., 2007), suggesting that the duration of deficiency in oestrogens is important. Similarly, in a large prospective study of over 15,000 women, a later age at menarche, younger age at menopause, shorter reproductive span and undergoing hysterectomy were all associated with an elevated risk of dementia (Gilsanz et al., 2019). However, evidence from oestrogens-based hormone replacement therapy (HRT) research remains equivocal. Numerous case-control studies in women have shown a reduction in AD risk and a delayed onset associated with HRT use and duration of use (Tang et al., 1996, Zandi et al., 2002), with a meta-analysis associating hormone use to reductions in dementia risk of nearly 40% (LeBlanc et al., 2001). However, in 2003 and 2004, results from two large clinical trials by the Women's Health Initiative showed that women who initiated HRT in the late postmenopausal stage (aged 65 years and above) experienced an increased risk of dementia and cognitive decline regardless of the type (natural or surgical) of menopause (Shumaker et al., 2004). It would appear that the timing of HRT administration relative to menopause onset is a key factor, with the weight of evidence suggesting that the neuroprotective or harmful effects of oestrogens depends on the age and stage of menopause at the time of initiation.

Oestrogens act in the central nervous system through various mechanisms, including neurotransmitter synthesis, receptor expression and membrane permeability**.** Oestrogens have been shown to modulate neurogenesis in the hippocampal formation in rodents (Sheppard et al., 2019), with all three oestrogen receptors (ERα, ERβ, and GPER1) found in the dorsal and ventral horns of the hippocampus and within the CA1, CA2, CA3, and dentate gyrus, in the nucleus and at extranuclear sites such as the dendrites (Mitra et al., 2003). It is unclear whether similar hormone effects would be found in humans, considering differences in rates of change of neurogenesis between the species (Sorrells, et al., 2018). Understanding how oestrogens affect structural plasticity of the hippocampus is crucial to understanding the functional outcomes of these modifications and to potential future treatments for hippocampal dysfunction, a key factor in diseases such as AD (Sheppard et al., 2019).

In premenstrual women, bilateral increases in medial temporal lobe (MTL) volume - particularly in the hippocampus - are found in the late relative to the early follicular phases, suggesting that the anatomy of this brain structure is sensitive to fluctuations in levels of oestrogens (Lisofsky et al., 2015). Moreover, women using hormonal contraceptives showed significantly larger parahippocampal gyri and temporal structures along with other cortical regions, relative to women not using contraceptives (Pletzer et al., 2010). Small scale studies have also shown that after controlling for age, postmenopausal women still have higher rates of hippocampal volume loss compared to premenopausal women (Kim et al., 2018, Mosconi et al., 2018). As before, though, evidence determining the effects of HRT from several small human case-control and cohort studies is mixed; some showing an increase in the volume of the MTL in HRT users relative to non-users (Boccardi et al., 2006, Erickson et al., 2005), other showing no group differences (Eberling et al., 2004, Resnick et al., 1998), or even a decrease in hippocampal volume in HRT users compared to a placebo group (Resnick et al., 2009).

The MTL is a key structure in cognition, particularly vulnerable to age-related decline and the first to show degeneration early in Alzheimer’s disease (AD) (Braak and Braak, 1997). It is noteworthy that women have a higher prevalence of AD compared to men, even after accounting for greater longevity (Vest and Pike, 2013). Moreover, in multiple transgenic mouse models of AD, female mice show greater neuropathology and behavioural impairments relative to males (Howlett et al., 2004, Lee et al., 2002), suggesting an increased vulnerability to AD pathogenesis in females. This vulnerability may be driven by both developmental sexual dimorphisms and differences in circulating and brain levels of sex steroid hormones oestrogens, progesterone, testosterone and their metabolites (Vest and Pike, 2013). Therefore, depletion of levels of oestrogens in postmenopausal women could increase risk for cognitive decline via anatomical changes in the MTL. These associations between oestrogens level and MTL anatomy and cognitive performance could well interact with the individual’s inherent genetic risk for AD and oestrogens level, both having shown the ability to predict hippocampal volumes. Polygenic score (PS) – the total added risk of common variants associated with a specific phenotype (Wray et al., 2014)– for AD risk has shown to predict hippocampal volume even in young healthy participants (Chauhan et al., 2015), and the same has been reported for the PS of oestradiol concentration in a sample of depressed men and women (Smeeth et al., 2019). Similarly, polygenic risk score for AD has been found to predict individual differences in rate of cognitive decline in normal aging (Kauppi et al., 2020). However, to date the potential interaction with hormone therapy has not been investigated.

The aim of this study was to investigate whether events affecting oestrogens exposure (i.e. age at menarche, age at menopause onset, exposure to HRT and age of first live birth) predicted the volume of brain structures within the MTL and/or performance on a prospective memory test in a large sample of healthy postmenopausal women from the UK Biobank cohort (www.ukbiobank.ac.uk). The prospective long-term memory task was used as an MTL-dependent task (Naya, 2016) based on its clear episodic long-term memory component (Burgess and Shallice, 1997). We were also interested in testing the potential interaction between any of the above significant predictors and polygenic scores for AD risk or oestradiol concentration, the most common form of oestrogen. In addition, we examined the highly interconnected structures that surround the hippocampus proper (entorhinal, perirhinal, and parahippocampal cortex, amygdala) that, together, comprise the MTL. It is known that MTL subregions are differentially impacted by chronological aging (Malykhin et al., 2017, Olsen et al., 2017) and display distinct patterns of neuropathology in AD (Wolk et al., 2017).

## 2 Methods

### 2.1 Sample

The UK Biobank is a large population-based cohort that includes 502,505 individuals recruited across the United Kingdom between 2006 and 2010 when aged 40–69. Extensive data has since been collected at different follow-up timepoints, including brain MRI scans from a subset of participants. For the purposes of this study, data from 10,924 postmenopausal women with completed structural MRI scans were included. Postmenopausal status was defined by self-report to the questions “Have you had your menopause (periods stopped)?”. Possible answers were “yes”, “not sure”, “no” and “prefer not to say”. Only those women answering “yes” were included in the sample. Age of menopause was determined by self-report to the question "How old were you when your periods stopped?". HRT status was determined by self-report to the following questions: “Have you ever used hormone replacement therapy (HRT)?”, “How old were you when you first used HRT?” and “How old were you when you last used HRT?”.

**Fig. 1:**
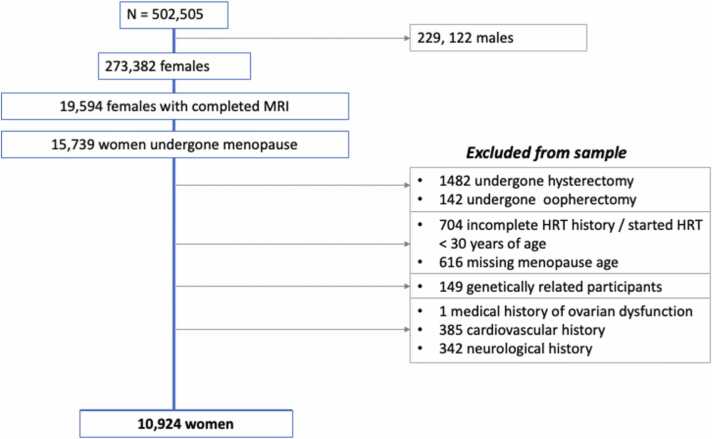
Case selection flowchart. HRT: hormone replacement therapy.

As shown in Fig. 1, we excluded women with a cardiovascular or neurological history (including those with chromosomal aberrations that could possibly impact menopausal age), those who had previously undergone a hysterectomy or oophorectomy, women who started menopause before age 30 and those with incomplete data regarding menopause age and HRT. Participants of non-British/Irish ancestry (defined as >4 × standard deviation from 1000 G phase 3 GBR sample mean based on first 3 principal components **–** 934 in total**)** were removed, as well as related individuals (estimated kinship coefficient > 0.0442 i.e. 3rd degree relatives, coefficient of relatedness > 12.5%). A comprehensive list of exclusion criteria is presented the Supplementary material.

Ethical approval to the UK Biobank was granted by the NorthWest Multi-Centre Ethics committee. Data were released under project reference 17044. Written informed consent was obtained from all participants. Raw data used in this study is available upon application from UK Biobank.

### 2.2 Polygenic score

Genotyping was conducted by Affymetrix for UK Biobank using bespoke Axiom arrays. Full details of the genotyping pipeline is openly available(Bycroft et al., 2018).

To calculate polygenic scores (PS) the imputed UKBB data were linkage disequilibrium (LD) pruned with PLINK (http://zzz.bwh.harvard.edu/plink/) by retaining the variant with the smallest p-value from each LD block and excluding variants with r2 > 0.1 in a 1000-kb window, using a random sample of 10000 individuals with ‘most probable genotypes’ computed from imputed allele ‘dosages’. Single nucleotide polymorphisms (SNPs) were removed if they had a low minor allele frequency < 0.01, low imputation quality score (INFOscore<0.4) and Hardy-Weinberg equilibrium p-value< 10^-6^.

The PS for AD risk was calculated based on a GWAS of clinically identified AD cases that importantly does not include participants from UK Biobank (Kunkle et al., 2019). The PS for oestradiol concentration was based on published summary statistics from a GWAS study on sex hormones (N = 2913) (Ruth et al., 2016). PS were calculated using PLINK with four different p-value thresholds (0.5, 0.1, 0.00001 and 5e-7). No oestradiol specific score was computed for the most stringent p-value threshold since no SNPs were retained. PS were adjusted considering the first 15 principal components for population stratification and the genotyping array and then standardised into Z-scores**.** As such our PS for AD represents the effect size weighted genome-wide count of common alleles associated with risk for AD at a given p-value threshold carried by an individual. Likewise, the PS for oestradiol represents the effect size weighted count of alleles associated with higher levels of oestradiol concentration.

### 2.3 Brain phenotypes

Brain images were acquired at three imaging centres equipped with identical scanners (Siemens Skyra 3 T, Siemens Healthcare, Erlangen, Germany) and a 32-channel head coil (Siemen’s Medical Solutions, Germany). High-resolution (1 mm isotropic voxel), T1-weighted, 3D magnetization-prepared gradient echo (MPRAGE) structural images (TI/TR = 880/2000 ms) were acquired as part of a longer MRI protocol (http://biobank.ctsu.ox.ac.uk/crystal/refer.cgi?id=2367).

Pre-processing and quality checking of images was undertaken by UK Biobank and followed a standardised and openly available pipeline (Alfaro-Almagro et al., 2018). Briefly, gradient distortion correction was applied, the brain is extracted using BET (Smith, 2002), registered to standard MNI152 space using FLIRT (Jenkinson, et al., 2002) and FNIRT, tissue-type segmentation is applied using FAST (Zhang et al., 2001). A comprehensive quality control pipeline determined a set of 190 features for use in a manually trained machine learning automated classifier to find any issues with the T1 structural images (Alfaro-Almagro et al., 2018). The quality control features include those that characterise subcortical structures, subcortical volumes and asymmetries between subcortical structures. The analysis presented in this paper was based on the Imaging Derived Phenotypes (IDPs) estimated using Freesurfer v6.0.0 (Elliot, et al., 2018), representing key imaging variables: total grey matter, hippocampal, temporal lobe and parahippocampal volumes; perirhinal and entorhinal cortex volumes and amygdala volume. As secondary analysis we also explored hippocampal subfield volumes (CA1, CA2/3, CA4 and Dentate gyrus).

For further quality control purposes, participants with excessive head motion in the scanner (determined during the fMRI scan sequence, UK Biobank data field 25741), defined as more than 3 interquartile ranges above the 75th percentile were excluded from analysis. As a result, 1458 statistical outliers were identified with excessive head motion and were removed from the analysis. Next, statistical outliers were defined in a similar manner for each brain region (more than 3 interquartile ranges either below the 25th percentile or above the 75th percentile) and were excluded from analysis. The mean number of outliers across brain regions identified and removed for regional MRI analysis was 9 (range across regions = 0–24).

### 2.4 Prospective memory

The results of a prospective memory task administered through a computerised touchscreen interface were used. The test was designed specifically for the UK Biobank to allow administration at scale without examiner supervision. The task shows evidence of an underlying performance factor and good stability over time (Lyall et al., 2016). Participants were asked to remember to perform a pre-planned instruction. Specifically, at the beginning of the test battery, they were presented with the following instruction: ‘At the end of the games we will show you four coloured symbols and ask you to touch the blue square. However, to test your memory, we want you to actually touch the orange circle instead’. If participants remembered to touch the orange circle on first attempt, they were coded as ‘correct’ (1), while those failing to do so were set to 0. The sample size for this task is reduced compared to the brain structure analysis (n = 10,319). As a secondary confirmative analysis, we examined another cognitive task implemented by UK Biobank that also partly taps into prospective memory: word-pairs. Analyses of this task have reduced statistical power due to a greater number of missing scores (n = 7313). In this task, participants were asked to memorise a list of word-pairs, after distraction by performing another unrelated cognitive test, participants were asked to recall the second word of each pair when presented with the first word. We analysed the number of correct responses to this task.

### 2.5 Statistical analyses

Statistical analyses were conducted in *R* (version 1.1.463). With respect to missing values, the data presented is a complete case analysis.

Multiple regression models were used for brain phenotype predicted variables and word-pairs, whereas logistic regression was used for prospective memory due to its bivariate (correct v incorrect) nature. In both cases predictor variables used were duration of HRT use, years from previous HRT use, age at HRT initiation, age at menarche, menopause onset age, and age at first live birth. Confounders included in both cases were participant age at the time of MRI scan, education, socioeconomic status (Townsend index). The linear mixed effects models for brain measures also included as confounders: hemisphere, normalised grey matter volume, MRI scan site, head motion from resting fMRI, and z-table position in the scanner, height and weight. In all cases, the predictors and confounders were centred to prevent errors in statistical inference. Multi-collinearity was tested by calculating the variance inflation factor (VIF, using ‘performance’ package version 0.6.0 in R) and all models showed low multicollinearity (VIF < 5).

To investigate the potential effects of the above predictors in interaction with PS for AD risk and oestradiol concentration, a second model was computed including an interaction term between any significant predictor from the above analyses and the aforementioned PS.

Resulting p-values were corrected for multiple comparisons using the false discovery rate (FDR) method via the ‘p.adjust’ function in the ‘stats’ package in R by considering all of the tests in each brain region; the corrected p-values are reported unless stated otherwise.

The 95% confidence intervals, ß values and adjusted p-values are reported for each regression model. Effect sizes reported correspond to the semi-partial (part) *R*^2^ values for any significant predictors, using the partR2 package in R.

## 3 Results

A total of 10,924 participants aged 47–81 years were included in this study. Baseline characteristics for the study population are presented in Table 1.

**Table 1:** Baseline characteristics. Mean ± standard deviation shown.

	Total Sample
*n*	10924
Age, years	63.41 ± 6.65
BMI, kg/m^2^	25.15 ± 4.46
Age completed education, years	17.22 ± 2.31
Menarche age, years	12.77 ± 2.41
Menopause age, years	50.83 ± 3.96
Townsend deprivation index	-1.98 ± 2.64
Age at first live birth, years	26.80 ± 4.51
History of HRT use, n (%)	2819 (25.8%)
HRT duration, years	6.61 ± 5.28
HRT initiation, years from menopause onset	-0.26 ± 2.75

BMI: body mass index; HRT: Hormonal Replacement Therapy

### 3.1 Menopause age and HRT duration predict medial temporal lobe volume (model 1)

Beta values, confidence intervals and corrected p-values are shown in Table 2 for each medial temporal lobe region and hormonal predictor variable. The R^2^ values for the full model fit can be found in Supplementary Table 1.

**Table 2:** Summary statistics from the linear mixed effect regression model (Model 1). P-values are FDR-adjusted for multiple comparisons. HRT initiation is measured in years relative to menopause onset.

	*Fixed effects in regression model*																		
	HRT duration				HRT initiation				HRT years from last use				Menopause Age				Reproductive Span		
	***b***	***95% CI***	***p***		***b***	***95% CI***	***p***		***b***	***95% CI***	***p***		***b***	***95% CI***	***p***		***b***	***95% CI***	***p***
Hippocampus	0.48	[− 2.75, 3.72]	0.770		2.00	[− 2.65, 6.64]	0.501		1.81	[− 0.29, 3.90]	0.443		6.08	[2.86, 9.31]	**0.0006**		5.72	[2.73, 8.71]	**0.0005**
Parahippocampal gyri	2.53	[0.66, 4.40]	**0.042**		2.65	[− 0.004, 5.34]	0.117		0.36	[− 0.86, 5.34]	0.700		4.17	[2.31, 6.04]	**0.00006**		4.19	[2.46, 5.92]	**0.00001**
Perirhinal cortex	-0.51	[− 2.72, 1.71]	0.770		2.92	[− 0.23, 6.08]	0.117		0.99	[− 4.42, 2.41]	0.443		2.56	[0.37, 4.75]	**0.0280**		2.95	[0.91, 4.98]	**0.0077**
Entorhinal cortex	-1.38	[− 4.38, 1.62]	0.616		5.62	[0.13, 9.90]	0.051		0.97	[− 0.97, 2.90]	0.547		0.54	[− 2.43, 3.51]	0.7207		1.34	[− 1.14, 3.79]	0.3415
Amygdala	-1.47	[− 3.24, 0.30]	0.260		-0.08	[− 2.62, 2.45]	0.949		0.23	[− 0.92, 1.37]	0.700		2.10	[0.33, 3.86]	**0.0280**		2.22	[0.59, 3.85]	**0.0099**
*Hippocampal subfield*																			
CA1	-0.19	[− 0.53, 0.14]	0.365		-0.04	[− 0.50, 0.45]	0.990		-0.002	[− 0.22, 0.21]	0.984		0.21	[− 0.12, 0.54]	0.2059		0.30	[− 0.007, 0.61	0.0563
CA2/3	-0.05	[− 0.19, 0.09]	0.365		0.00	[− 0.20, 0.20]	0.924		-0.02	[− 0.11, 0.07]	0.598		0.11	[− 0.024 0.23]	0.1648		0.13	[0.004, 0.26]	0.0563
CA4	-0.07	[− 0.19, 0.05]	0.514		0.07	[− 0.10, 0.25]	0.990		0.04	[− 0.04, 0.12]	0.888		0.15	[0.03, 0.27]	**0.0366**		0.17	[0.06, 0.28]	**0.0106**
Dentate gyrus	-0.11	[− 0.26, 0.03]	0.365		0.08	[− 0.13, 0.28]	0.924		0.06	[− 0.03, 0.15]	0.598		0.18	[0.03, 0.31]	**0.0366**		0.19	[0.06, 0.32]	**0.0106**

b: beta; 95%CI: 95% confidence interval; p: FDR-adjusted significance level; HRT: Hormonal replacement therapy

Accounting for all other variables in model 1, the duration of HRT use was found to predict grey matter volume selectively in the parahippocampal gyri, with longer use associated with increased volume (2.53 mm^3^ per years used, p_FDR_ = 0.042, partial R^2^ = 0.0014). The duration of HRT was not predictive of volume in any other brain region (all p_FDR_ > 0.05).

**Fig. 2:**
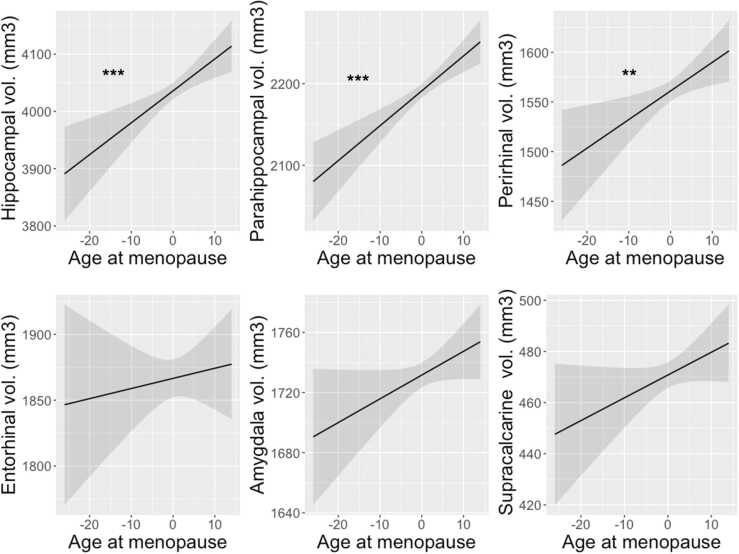
Predicted values (marginal effects) of age of menopause onset on medial temporal lobe volume in post-menopausal women from a linear mixed effect model, after accounting for all other variables, with 95% confidence intervals. The x-axis shows the de-meaned age at menopause (mean menopause age = 50.8 years). * ** p < 0.001, * p < 0.05 after adjusted for multiple comparisons. Hippocampal subfield ROIs y-axis shown in red. DG: dentate gyrus. Vol.: volume.

As seen in Fig. 2, menopause age was found to predict grey matter volume in the hippocampus (6.08 mm^3^ per year, p_FDR_ = 0.00057, partial R^2^ = 0.0032), parahippocampal gyri (4.17 mm^3^ per year, p_FDR_ = 0.00006, partial R^2^ = 0.0038) perirhinal cortex (2.56 mm^3^ per year, p_FDR_ = 0.028, partial R^2^ = 0.0010) and amygdala (2.10 mm^3^ per year, p_FDR_ = 0.028, partial R^2^ = 0.0008), with earlier menopause age associated with lower volume.

To assess whether the effect of menopause age on MTL volume was driven by reproductive lifespan, analyses were repeated with reproductive span (menopause age – age at menarche) included and both menopause age and menarche removed, due to multicollinearity. As with menopause age, reproductive span predicted grey matter volume in the hippocampus (p_FDR_ = 0.0005, partial R^2^ = 0.0031), parahippocampal gyri (p_FDR_ = 0.00001, partial R^2^ = 0.0044), perirhinal cortex (p_FDR_ = 0.0077, partial R^2^ = 0.0016) and amygdala (p_FDR_ = 0.0099, partial R^2^ = 0.0009), with a longer reproductive span associated with greater tissue volume (see Supplementary Figure 1).

The number of years since having last used HRT, and the time of HRT initiation relative to menopause onset was not predictive of grey matter volume in any region.

In an analysis of hippocampal subfields, both menopause age and reproductive span were predictors of grey matter volume in CA4 and the dentate gyrus (see Table 2 and Fig. 2).

### 3.2 Polygenic score for AD and oestradiol concentrations (model 2)

There was no effect of either AD risk or oestradiol PS on hippocampal, parahippocampal gyri, amygdala and perirhinal cortical volume, after accounting for the other predictors in the model (all pFDR > 0.05) at any of the p-thresholds used. Similarly, there was no significant interaction between menopause age and either AD or oestradiol PS for hippocampal, parahippocampal gyri and perirhinal cortical volume after adjusting for multiple comparisons. Similarly, there was no interactive effect of PS (AD and oestradiol) and either menopause age or HRT duration on volume in any of the regions (all pFDR > 0.05) at any of the p-thresholds used.

The observed effect of menopause age on hippocampal, parahippocampal and perirhinal cortical volume remained when AD PS and oestradiol PS were added to the model (all p_FDR_ < 0.05). Similarly, the effect of HRT duration of parahippocampal volume remained when PS was added to the model (p_FDR_ < 0.05). Oestradiol PS did not predict menopause age (all p > 0.05).

### 3.3 Prospective memory performance and menopause onset age (model 1 & 2)

Increased menopause age had a positive effect on performance on the prospective memory task (ß = 0.050, 95% confidence intervals 0.008, 0.091, p = 0.019). There was no effect of HRT (duration, initiation age, years since use) on prospective memory (all p > 0.05). As above, we then conducted an exploratory analysis by calculating reproductive span and repeating the analysis with all factors in model 1 included, except for age at menopause and menarche because of multicollinearity. Similar to menopause age, a longer reproductive span had a positive effect on performance on the prospective memory task (ß = 0.050, 95% confidence intervals 0.011, 0.089, p = 0.011). Likewise, increased menopause age showed a trend to positively predict performance in the word-pairs task (ß = 0.024, 95% confidence intervals −0.002, 0.052, p = 0.07), this association reaching significance for reproductive span (ß = 0.026, 95% confidence intervals 0.001, 0.051, p = 0.04). There was again no effect of HRT (duration, initiation age, years since use) on word-pairs task performance (all p > 0.05).

For model 2, there was no significant interaction between menopause age and either AD risk or oestradiol PS for prospective memory or word-pairs after adjusting for multiple comparisons (all p_FDR_ > 0.05).

## 4 Discussion

We used a large British population cohort of women with a mean age of 63 (range 45–80) who reported to be post-menopausal and were free of known neurological and cardiovascular disease to investigate the complex relationship between reproductive factors, neuroanatomy and cognition. The high statistical power associated with our large sample allowed us to detect potential small associations that might have escaped previous studies with more modest sample sizes, and to explore issues relating to the timing of reproductive events and hormone replacement, and genetic predispositions, whilst controlling for multiple key confounds. As such, our results complement previous findings from better controlled studies with purposely targeted smaller samples. Given that both age of menopause and age of menarche have been shown to be in part influenced by malleable environmental factors(Ceylan and Ozerdogan, 2015) (e.g. physical activity, diet, use of tobacco and alcohol, body mass index), the presence of any significant associations between reproductive span and brain health following menopause has implications for health promotion in early life to improve brain and cognitive ageing later in life.

We found hippocampal volume (along with some MTL structures) to be larger and the recall on prospective memory better, in women with later age of menopause. The same relationship was found for women with a longer reproductive span. A secondary analysis exploring hippocampal subfields showed larger volumes of CA4 and the dentate gyrus in women with a longer reproductive span and later menopause. Finally, women who had taken hormone replacement therapy for longer durations were found to have larger parahippocampal cortices, independent of when they had initiated HRT relative to menopause, and the number of years since they had last used HRT. Polygenic risk for AD and for oestradiol concentrations did not predict MTL volume and did not interact with menopause age or HRT duration to have an effect on brain structure, suggesting that the above associations are independent of the participants’ genetic predisposition to AD or oestradiol concentrations. Recent work using the same database has shown an association between AD polygenic risk and hippocampal volume specifically (Tank et al., 2022), however these studies were not restricted to postmenopausal females and thus had a much larger sample size, without accounting for hormone factors, HRT-related factors or education in their statistical modelling, which plausibly explains the difference in results.

Our results suggest that the duration of HRT use has a small positive effect on parahippocampal gyri volume, whereas HRT use was not found to significantly impact total hippocampal volume nor other medial temporal lobe structures in post-menopausal women. Short-term estradiol administration over 3 months in post-menopausal women has shown an increase in hippocampal grey-matter volume, but only in those receiving a larger dose of 2 mg estradiol compared to 1 mg estradiol and placebo (Albert et al., 2017). In our study, although longer term HRT appeared to have a positive effect on parahippocampal volume, this was found to be small, with HRT duration accounting for < 1% of the variance in brain structure.

The positive association reported here between later menopause age, and longer reproductive span with MTL structures and MTL-related cognition (Gordon et al., 2011) is consistent with the direction of effects seen more generally in the research literature: a later age at natural menopause and longer reproductive span has been observed to reduce risk of all-cause mortality, cardiovascular mortality, cardiovascular disease (Kim et al., 2015, Muka et al., 2016, Shadyab et al., 2017) and dementia (Gilsanz et al., 2019). Naturally high estradiol levels have been shown to confer an advantage on hippocampal-dependent spatial memory task (Patel, et al., 2022). A comparison between postmenopausal women on HRT versus an age-matched group not on HRT showed significantly larger hippocampal volume and other MTL structures in the HRT group (Kim, et al., 2023). A recent study on women with early midlife bilateral salpingo-oophorectomy (BSO) showed reduced hippocampal integrity specific to a BSO group not taking hormone therapy, with significantly smaller dentate gyrus/CA2/CA3 volumes which were associated with poorer scene recognition memory (Gervais, et al., 2022). One of the potential mechanism by which menopause age and reproductive span could influence MTL structure and MTL-related cognition that warrants further investigation, would be via a reduction in vascular risk factors, resulting in improved cognition and biological ageing (Forte and Casagrande, 2020). This interpretation is based on increasing evidence that exposure to oestrogens is cardioprotective and that cardiovascular and neuro-cognitive systems do not operate in isolation (Forte and Casagrande, 2020).

Despite the fact that our analyses controlled for the effects of body weight, we were unable to capture other vascular risk factors to test the above hypothesis. Notably, our cohort were healthy women with no history of cardiovascular disease, and effect sizes were small, with both menopause age and reproductive span individually accounting for less than 1% of the variance in MTL structure, whereas all the fixed factors combined in the model (i.e., the marginal effects, not including the random effects) accounted for between 10% and 57% of the variance. Further investigation is needed to determine if effect sizes are larger, potentially with clinical implications, in postmenopausal women with a history of cardiovascular disease.

Menopause age and reproductive span are influenced by a range of genetic and environmental factors (Karapanou and Papadimitriou, 2010). Our results suggest a link between hormonal factors and MTL volume; however, we found genetic factors such are common genetic risk for AD or common genetic predisposition to high oestradiol concentrations do not influence this link. Although these results focus on polygenic score for oestradiol concentration, progesterone also plays an important role and is commonly a component of HRT. Evidence suggests that AD pathogenesis is regulated by both oestrogens and progesterone in females (Vest and Pike, 2013) and that progesterone dynamically changes MTL volumes across the human menstrual cycle (Taylor et al., 2020). Thus, the potential positive role of progesterone in female ageing and hormone therapy use requires further attention.

This study has several limitations. Firstly, caution has been advised when using automatic segmentation of isotropic 1x1x1mm^3^ T1-weighted images to delineate hippocampal subfields (Olsen et al., 2019; Wisse et al., 2021) and therefore our secondary analysis regarding this segmentation should be regarded with caution, putting the focus on our whole hippocampus results. However, it is important to also note that a comprehensive automatic quality control procedure is implemented in UK Biobank that checks for issues with the T1-weighted images including features that characterise subcortical structures, subcortical volumes and asymmetries between subcortical structures (Alfaro-Almagro et al., 2018); and that we subsequently excluded imaging outliers to further control for the potential effect of extreme values resulting from deficient segmentation. If issues remain present, for datasets at this scale it is expected that poor segmentations can only add noise to the subfield IDPs and won’t systematically create biased results. Therefore, despite the caution, our results regarding hippocampal subfields should not be completely disregarded. Secondly, both the ages at menarche and menopause were reliant on self-reported recall of past events that could not be verified with medical records, which could result in misclassification. The STRAW+ 10 criteria (Harlow, et al., 2012) defines the postmenopausal period as being at least 12 months since final menstruation. Since self-reports in the UK Biobank data could be inaccurate, it may be the case that some of our participants were in the perimenopause stage. However, due to the age of our sample (86.6% over 55) and that we excluded those reporting to be unsure about their menopause state, participants potentially in perimenopause are likely to be a minority. To further explore whether potentially misclassified participants could have affected our results we repeated our analyses with a reduced sample where we only included participants aged over 55 and that already reported their periods to have stopped during a UK Biobank baseline assessment taking place at least 5 years earlier (on average 8 years before the scanning visit). Results remained similar to those of our full sample, suggesting that any menopause misclassification is unlikely to play a major part in the results reported here. Thirdly, although our statistical model was comprehensive in terms of the large number of hormonal, genetic, social and demographic factors accounted for, we were not able to account for blood pressure due to significant amount of missing data, and historical and current lifestyle factors such as smoking and exercise behaviours were not included to avoid overfitting the statistical models. Moreover, it was not possible to assess the nature (oestrogen only vs. oestrogen-progesterone) or route of HRT administration, despite there being metabolic differences between oral and transdermal routes (Stevenson et al., 1993). Future large-scale studies should consider acquiring this information to shed further light on these findings. Finally, driven by the available GWAS results in which we based the calculation of polygenic scores and the ethnic composition of the UK Biobank cohort clearly biased towards European individuals, our sample was limited to women of British/Irish ancestry. This limits the generalisability of our results, a problem that should be resolved as trans-ancestry genetic research becomes available.

## 5 Conclusion

A small but significant effect of menopause age and reproductive lifespan on medial temporal lobe volume and cognitive performance was observed in healthy postmenopausal women, with no evidence of a detrimental effect of HRT on key brain structures implicated in later life dementia. Further studies are needed to elucidate lifestyle, genetic, and environmental factors associated with menopause and reproductive lifespan to test whether vascular risk factors may mediate the link, with implications for healthy ageing and cognition.

## Funding and disclosure

This research was funded in whole, or in part, by the 10.13039/100004440Wellcome Trust [WT200804, WT224267]. For the purpose of open access, the author has applied a CC BY public copyright licence to any Author Accepted Manuscript version arising from this submission.

## CRediT authorship contribution statement

**Jessica J Steventon**: Conceptualization, Methodology, Formal Analysis, Writing – Original Draft, Funding acquisition, Visualization; **Thomas M. Lancaster**: Software, Formal Analysis; **Emily Simmonds Baker**: Data Curation, Software, Formal Analysis; **Matthew Bracher-Smith**: Data Curation, Software; **Valentina Escott-Price P**: Validation, Resources, Supervision, Writing – Review & Editing; **Katherine S. Ruth**: Conceptualization, Resources, Formal Analysis; **William Davies**: Conceptualization, Writing – review & Editing; **Xavier Caseras**: Conceptualization, Resources, Data Curation, Writing – Review & Editing, Supervision; **Kevin Murphy**: Conceptualization, Methodology, Validation, Writing – Review & Editing, Supervision, Funding acquisition, Project administration.

## Declaration of Competing Interest

The authors report no conflicting interests.
