# Supplementary material for: Menopause age, reproductive span and hormone therapy duration predict the volume of medial temporal lobe brain structures in postmenopausal women
Source: Psychoneuroendocrinology. 2023 Dec;158:106393. doi: 10.1016/j.psyneuen.2023.106393 (PMC13369427; doi:10.1016/j.psyneuen.2023.106393)
Supplement: Supplementary file 1 — Supplementary material [file mmc1.docx]

# Supplementary Materials

## Comorbid exclusion criteria

Self-reported and hospital admissions data were used to exclude participants based on comorbidities. For hospital admissions, both main and secondary diagnosis were used, and the ICD-10 disease coding is used. Women with ovarian Dysfunction E28 were excluded.

Cardiac conditions included in exclusion criteria:

- Cardiovascular disease: acute myocardial infarction (I21), subsequent acute myocardial infarction (I22), other acute ischaemic heart diseases (I24), chronic ischaemic heart disease (I25); heart failure (I50), cerebral infarction (I63). stroke, not specified (I64), vascular symptoms CVD (G46), subarachnoid haemorrhage (I60), Intracerebral haemorrhage (I61), Other nontraumatic intracranial haemorrhage (I62), aortic aneurysm and dissection (I71), other cerebrovascular diseases (I67), cardiac arrest (I46)
- Cardiomyopathy (I42)
- Occlusion: Occlusion and stenosis of precerebral arteries, not resulting in cerebral infarction (I65), Occlusion and stenosis of cerebral arteries, not resulting in cerebral infarction
- Conduct disorder: Atrioventricular and left bundle branch block (I44), Other conduction disorders (I45)

Neurological conditions included in exclusion criteria:

- Intracranial injury
- Dementia
- Brain pathology
- Encephalitis
- Epilepsy
- Hydrocephalus
- Multiple Sclerosis
- Meningitis
- Motor neuron disease
- Neurodegenerative disease
- Myasthenia gravis
- Parkinson’s Disease
- Paralysis
- Stroke

In addition, participants with chromosomal aberrations were excluded.


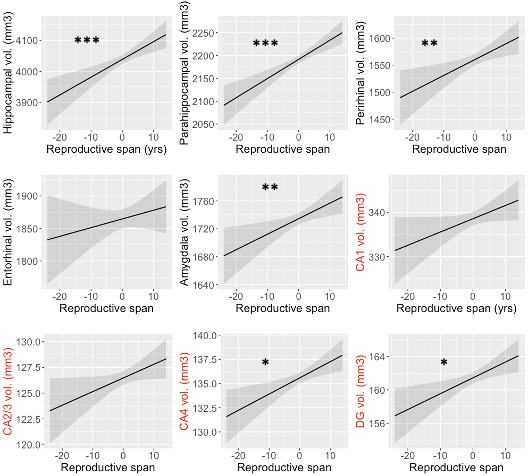


Supplementary Figure 2 Predicted values (marginal effects) of reproductive span on medial temporal lobe volume in post-menopausal women from a linear mixed effect model, after accounting for all other variables, 95% confidence intervals shown. *** p < 0.001, ** p < 0.01, * p < 0.05 after adjustment for multiple comparisons. DG: dentate gyrus. Hippocampal subfield y-axis labelled in red.

Supplementary Table 1. Pseudo R^2^ values for Model 1 using the approach of Nakagawa and Schielzeth^71^. Fixed R^2^ refers to marginal R^2^ values, representing the variance explained by fixed factors, whilst Fixed + Random refers to conditional R2 which is the variance explained by both fixed and random factors (i.e., the entire model). Equations for calculations can be found in https://www.rdocumentation.org/packages/MuMIn/versions/1.40.4/topics/r.squaredGLMM

| ***Dependent variable in Model 1*** | ***Fixed R^2^*** |  | ***Fixed + random R^2^*** |
| --- | --- | --- | --- |
| Hippocampus | 0.131 |  | 0.849 |
| Parahippocampal gyri | 0.189 |  | 0.723 |
| Perirhinal cortex | 0.10 |  | 0.530 |
| Entorhinal cortex | 0.566 |  | 0.767 |
| Amygdala | 0.393 |  | 0.797 |
|  |  |  |  |
| ***Hippocampal subfield analysis*** | |  |  |
| CA1 | 0.194 |  | 0.790 |
| CA2/3 | 0.180 |  | 0.706 |
| CA4 | 0.132 |  | 0.753 |
| Dentate gyrus | 0.177 |  | 0.778 |
